# P21-Activated Kinase 1: Emerging biological functions and potential therapeutic targets in Cancer

**DOI:** 10.7150/thno.46913

**Published:** 2020-08-01

**Authors:** Dahong Yao, Chenyang Li, Muhammad Shahid Riaz Rajoka, Zhendan He, Jian Huang, Jinhui Wang, Jin Zhang

**Affiliations:** 1 School of Pharmaceutical Sciences, Health Science Center, Shenzhen Technology University, Shenzhen, 518060, PR China.; 2 School of Pharmaceutical Sciences, Health Science Center, Shenzhen University, Shenzhen, 518060, PR China.; 3 State Key Laboratory of Biotherapy and Cancer Center, West China Hospital, Sichuan University, and Collaborative Innovation Center of Biotherapy, Chengdu 610041, China.; 4 Department of Medicinal Chemistry and Natural Medicine Chemistry, College of Pharmacy, Harbin Medical University, Baojian Road 157, Nangang District, Harbin 150081, PR China.

**Keywords:** PAK1, structure, cancer, targets, resistance, small molecular inhibitors

## Abstract

The p21-Activated kinase 1 (PAK1), a member of serine-threonine kinases family, was initially identified as an interactor of the Rho GTPases RAC1 and CDC42, which affect a wide range of processes associated with cell motility, survival, metabolism, cell cycle, proliferation, transformation, stress, inflammation, and gene expression. Recently, the PAK1 has emerged as a potential therapeutic target in cancer due to its role in many oncogenic signaling pathways. Many PAK1 inhibitors have been developed as potential preclinical agents for cancer therapy. Here, we provide an overview of essential roles that PAK1 plays in cancer, including its structure and autoactivation mechanism, its crucial function from onset to progression to metastasis, metabolism, immune escape and even drug resistance in cancer; endogenous regulators; and cancer-related pathways. We also summarize the reported PAK1 small-molecule inhibitors based on their structure types and their potential application in cancer. In addition, we provide overviews on current progress and future challenges of PAK1 in cancer, hoping to provide new ideas for the diagnosis and treatment of cancer.

## Introduction

The p21-activated kinases (PAKs) belong to the family of serine-threonine kinases and have been identified as effectors of Cdc42 and Rac1 small GTPases 1. PAKs are closely associated with a wide variety of cellular functions, including cytoskeletal motility, apoptosis, and cell cycle regulation, mainly by a various substrate phosphorylation 2. The PAK family consists of two subgroups; a subgroup I (PAK1, PAK2, and PAK3) and subgroup II (PAK4, PAK5, and PAK6) having display distinct as well as overlapping functions and are regulated by different mechanisms 3. The distinct roles of PAK family members in normal tissue development have been investigated by using gene knockout mouse models, with the phenotypes ranging from having no apparent effect to early embryonic death. In mice, *PAK1*^ (-/-)^ and *PAK5*^ (-/-)^ null mice are viable and healthy, whereas loss of *PAK2* or *PAK4* could cause embryonic lethality, and *PAK3*^ (-/-)^ would result in learning and memory defects 4. PAKs' dysregulation is involved in cellular homeostasis and functions implicated in a number of human diseases, including cardiac disorders, neurological disorders, and cancers 5, 6. Amongst the PAK family members, PAK1 and PAK4 are the most studied in human cancers, due to their central roles in many oncogenic signaling pathways, and they have emerged as potential therapeutic targets in cancer 7. Because PAK4 has been well summarized, including its signaling, regulation, and specificity 8, here, we focus our discussion on PAK1 in cancer.

*PAK1* gene amplification or protein overexpression was observed in many kinds of tumors, including ovarian cancer, breast cancer, colorectal cancer, and hepatocellular carcinoma 9-11. PAK1 overexpression has been identified as a diagnostic biomarker of overall survival and disease-specific survival in solid tumors patients 12. Furthermore, the role of PAK1 in leukemia has attracted more and more attention recently 13, 14. PAK1 acts as a protector in DNA-damage response caused by genotoxic therapeutic agents or radiotherapy via directly phosphorylating microchidia CW-type zinc finger 2 (MORC2-Ser739) and γH2AX 15. PAK1 dysregulation has been documented to be closely associated with cancer cell proliferation, metastasis, and drug resistance, and it has emerged as a promising target for cancer treatment 16. Many PAK1 inhibitors have been developed as potential preclinical agents for cancer therapy 17. In this paper, we review PAK1's roles in cancer, including its structure and autoactivation mechanism; its essential function from the onset, progression to metastasis, and even drug resistance in cancer; endogenous regulators; and cancer-related pathways. We also discuss the suitability of PAK1 as an anti-cancer drug target and recent advances in the development of PAK1 inhibitors based on their structure types. Furthermore, we provide our perspective on current advances and future challenges of PAK1 in cancer.

## Structure and the autoactivation mechanism of PAK1

**Figure 1:**
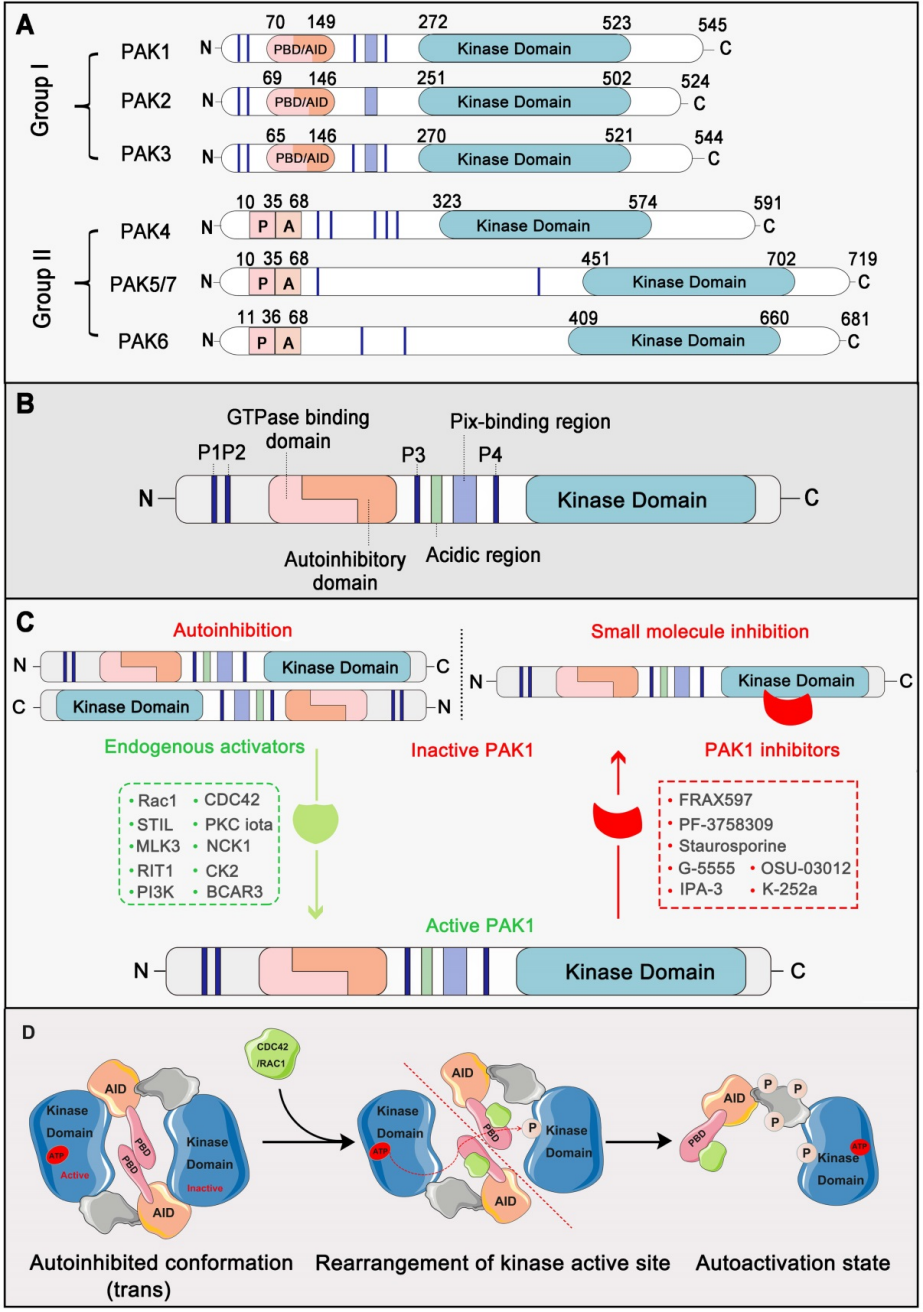
** PAK1 structure and the autoactivation mechanism.** (**A**) Schematic representation of the P21-activated kinases including Group I PAKs and Group II PAKs. Of them, PAK1-3 are divvied into Group I PAKs and PAK4-7 are divvied into Group II PAKs. (**B**) PAK1 contains a p21-binding domain (PBD) at the N-terminus for GTPase association, an auto inhibitory domain (AID) and a C-terminal kinase domain. (**C**) PAK1 can be activated or inhibited by numerous regulators and inhibitors. (**D**) The autoactivation mechanism of PAK1 which mainly dependent on phosphorylation.

PAKs belong to the STE20 family of serine/threonine kinases, which is comprised of group I (PAK1, PAK2, and PAK3) and group II (PAK4, PAK5, and PAK6) based on sequence and structural homology 18. Structurally, all six members contain a p21-binding domain (PBD) at the N-terminus for GTPase association, an autoinhibitory domain (AID), and a C-terminal kinase domain 19. The regulatory domains of groups I and II are structurally distinct, resulting in a different activation mechanism. For group I PAKs, the PBD domain overlaps with the AID domain. In contrast, group II PAKs only carry an AID-like pseudosubstrate sequence that inactivates the kinase activity of the Cdc42-bound PBD domain 3. PAK1 is the most extensively studied member of the group I PAKs, which is comprised of 545 residues, including a GTPase-binding region (residues 75-105), autoinhibitory domain (residues 70-149), and kinase domain (residues 272-523) 20 (**Figure 1A, 1B**). Interestingly, the autoactivation mechanism of PAK1 occurs via an unusual dimerization autoinhibitory to a multi-stage activation switch 21. For the initial state, the PAK1 dimer is comprised of two PAK1 molecules in an asymmetric antiparallel manner (or face to face); one monomer adopts an active conformation, and the other is inactive 22. The PBD domain overlapping with the AID domain occupies the cleft of the kinase domain of another PAK1 monomer and stabilizes a disabled catalytic site. Subsequently, the binding of an activated endogenous activator, such as Cdc42 and Rac, to the PBD initiates the interactions with the proximal amino acids and phosphoinositide, which disrupts the dimer and causes distinct changes in the conformation of the catalytic domain, resulting in the dissociation of AID domain from the kinase domain 23. As a result, the activation loop is released and the unique Thr423 of the inactive monomer is phosphorylated via a trans-phosphorylation as the conventional substrate of another active monomer, which is very important for the full catalytic activity of PAK1 24. Once Thr423 has been phosphorylated, PAK1 can autophosphorylate at several sites (phosphoserine) within the first 250 amino acids, which could prevent the kinase from reverting to an inactive conformation (**Figure 1C, 1D**). By contrast, group II PAKs lack a defined inhibitory segment and are active in the absence of small GTPases. It is worth noting that phosphorylation can not only regulate the activity of PAK1, but also the key to PAK1 regulating various proteins to participate in various life processes of cancer 25.

## PAK1 and cancer

**Figure 2:**
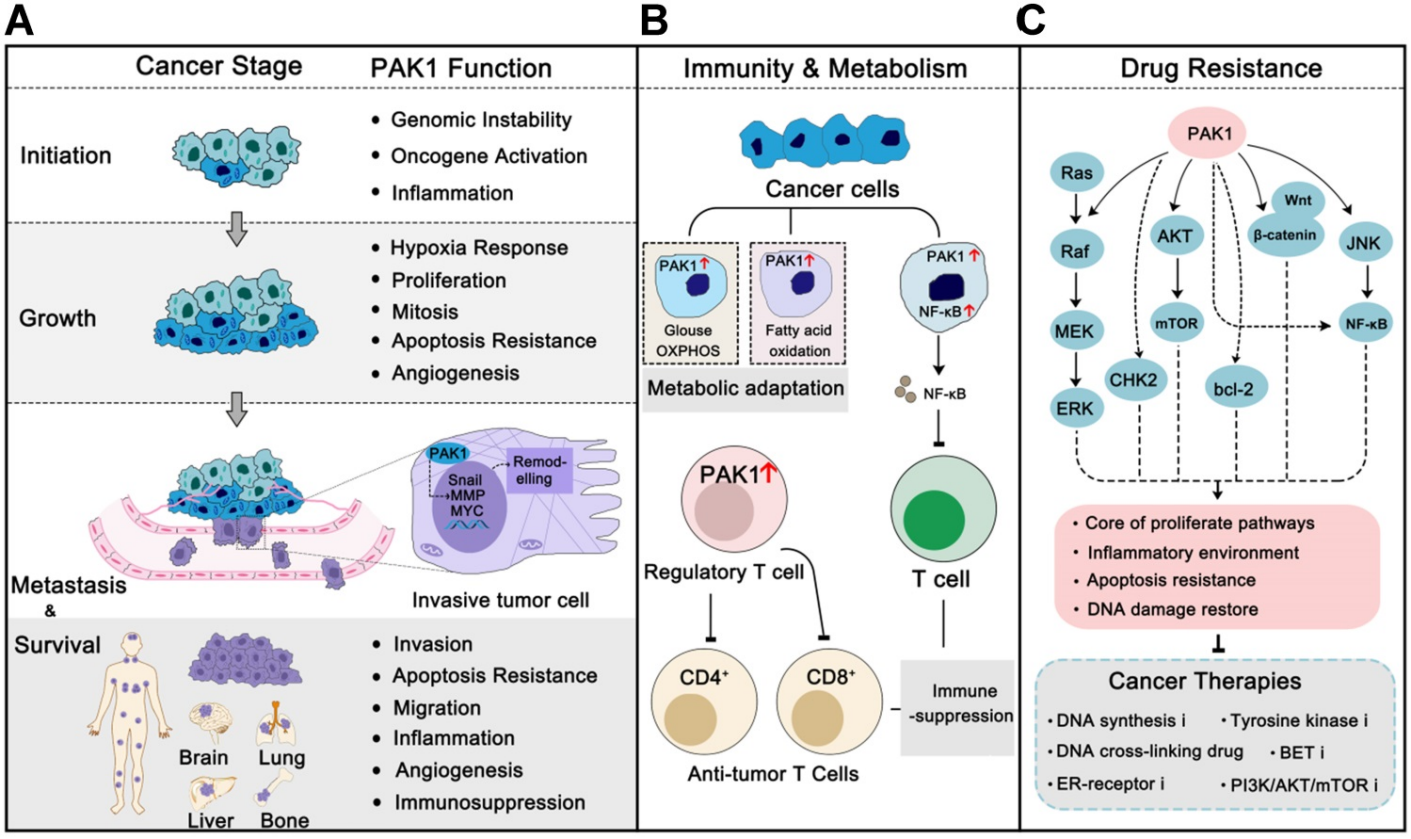
** PAK1 in cancer progression, immunity, metabolism and drug resistance.** (**A**) In different cancer stages, PAK1 functions properly to promote cancer progression, including regulation of cancer-related pathways, angiogenesis, tumor microenvironment remodeling, immune evasion, and apoptosis inhibition. Similarly, these effects also contribute to drug resistance in cancer therapies. (**B**) PAK1 can help cancer cells change their metabolic pattern to adapt to new survival environment. In addition, PAK1 can affect the vitality of immune cells and help cancer cells immune escape. (**C**) PAK1 promotes the resistance of cancer cells to various anti-tumor drugs through the PAK1 PPI network.

PAK1 is frequently overexpressed or excessively activated in almost all cancer types, and it is more pronounced in malignancies 26. For instance, PAK1 was found to be highly amplified and phosphorylated in breast cancer, which is the cumulative consequence of multiple cancer-related mutations, such as *PIK3CA* and *TP53*, and contributes to maintaining the fitness and survival of cancer cells under multiple pressures 27, 28. Studies of the relationship between PAK1 and cancer were started in the mid-1990s. It was first reported that PAK1 was involved in the MAPK signal transduction in 1995 29 and regulated actin cytoskeleton remolding in 1997 30. Subsequent studies revealed that PAK1 participated in cell migration and motility 31. PAK1 was first confirmed to be involved in breast cancer cell proliferation and cancer progression in 2000 32. Over the last 20 years, researchers have increasingly recognized the role of PAK1 in the progression of cancer. PAK1 is now involved in almost every stage of the cancer process, from onset to progression to metastasis, and even drug resistance 33, 34 (**Figure 2**). PAK1 is also considered a cancer hallmark that modulates cancer therapeutic outcomes 14, 34. Here, we review the function and regulation of PAK1 in cancer.

### PAK1 in cancer initiation

The cancer initiation is the accumulation of single or multiple gene mutations, the aberrant interactions of different signaling, and the result of cancer cells proliferating and surviving in the microenvironment 35. Oncogenic transformation requires global fine-tuning of oncogene-regulated signals, including the rearrangement of the actin cytoskeleton, response to the environment, immune evasion, and eventual survival. PAK1 is one of the central nodes in this complex oncogenic signaling pathway network, and it can promote oncogenic transformation in a variety of ways 36. Functionally, PAK1 is an oncogene that when overexpressed or excessively activated plays an important role in tumorigenesis 27. When the tumor initially forms, it suffers relatively low survival pressure because oxygen and nutrients are relatively abundant. The activation of PAK1 allows it to participate in cancer cell proliferation pathways to inhibit apoptosis and facilitate the survival of cancer cells. Indeed, recent studies have been shown that PAK1 inhibition impeded carcinogenesis in several tumors, such as breast cancer, intestinal tumor, lung cancer, and melanoma 37-39. In addition, due to the effect of PAK1 on chromatin remodeling 40, many oncogenes are activated and then participate in the tumorigenesis process 41. Elsewhere, it was found that PAK-dependent microchidia CW-type zinc finger 2 (MORC2) phosphorylation promoted DNA repair to maintain cancer cell fitness 42. And in gastric cancer, high phosphorylation of MORC2 is always accompany with high expression of PAK1 and involves in poor prognosis 43. Intriguingly, although cancer-associated mutations are often hereditary, not all cancer-causing mutations develop into cancer, and they also require the remodeling effect of the tumor microenvironment. Therefore, it is not surprising that the PAK1-induced inflammatory microenvironment is another accomplice in the process of tumorigenesis 44. PAK1-regulated STAT3-NF-κB signaling continues to activate the inflammatory response, and the releases of IL-1α and IL-18 will induce cancer cell proliferation, survival, and immunosuppression. Collectively, PAK1 activation makes cancer cells more robust and invisible to the immune system, thus paving the way for further proliferation.

### PAK1 in cancer growth and angiogenesis

Cancer cells must grow into a sufficiently large population before they can spread and metastasize. These cancer cells are just like a small social group, each with a division of labor and always ready for further progression 35. Cancer cells are often in a state of hypoxia and nutrient deficiency due to their uncontrolled rapid proliferation. Hypoxia presents both challenges and opportunities for tumors. In principle, hypoxia is not conducive to the proliferation and survival of cancer cells, but the hypoxia response will exacerbate the instability of tumor genome and microenvironment 45. PAK1 can stimulate the activation of hypoxia-inducible factors (HIFs) to promote the adaptation of tumor cells to hypoxic conditions and further induce metabolic reprogramming of tumor cells 46. On the one hand, PAK1 takes part in HER2, EGFR, MAPK, PI3K/AKT, and Wnt/β-catenin pathways and properly coordinates these signals to ensure tumor cell proliferation under hypoxic conditions 37. On the other hand, PAK1 accelerates angiogenesis in the tumor microenvironment to ensure energy supply. In this process, PAK1 can assist with basic fibroblast growth factor (bFGF) - and vascular endothelial growth factor (VEGF)-mediated Raf-1 and MEK1 activation to escape from apoptosis during angiogenesis 47. PAK1 regulated HIFs, p38, Akt and NF-κB activation also contributes to angiogenesis 48, 49. Besides, regulation of cell cycle progression, apoptosis resistance, and immune escape; PAK1 also creates favorable conditions for the formation of tumor society. Collectively, PAK1 activation contributes to the tumor society's stability and progress.

### PAK1 in cancer metastasis and survival

One of the most studied biological functions of PAK1 is their contribution in cancer metastasis. Cancer metastasis is an extremely complex process involving multiple chronological stages. In simple terms, it includes local invasion and infiltration into blood vessels, the survival of tumor cells in circulation, extravasation to secondary areas, and metastasis to new tissues and organs 50. PAK1 can participate in almost every stage of the transfer process. First, tumor cells must break through the underlying basement membrane of the tumor, namely, the degradation of the extracellular matrix. This process requires the participation of proteolytic enzymes, and PAK1 can promote tumor epithelial-mesenchymal transformation (EMT) by up-regulating the expression of matrix metalloproteinase genes and activating transcription factors, such as snail and twist 26, 48. In addition, cancer cell metastasis must undergo a signal-dependent cytoskeleton remodeling process. It is worth noting that PAK1 was reported to exhibit the function of cytoskeletal regulation in 1997 30. Increasingly, studies have shown that many proteins involved in cytoskeleton formation and regulation are the phosphorylated substrates of PAK1, including the microtubule-destabilizing proteins stathmin 51, filamin 52, and the actin-binding proteins LIM-kinase (LIMK) 53, p41-Arc subunit of human Arp2/3 complex 54, and tubulin cofactor B (TCoB) 55. PAK1 regulates these substrates to maintain the flexibility of the cytoskeleton and to enable cells to migrate. Interestingly, RUN and FYVE domain containing 3 (RUFY3), which can be up-regulated by PAK1, localizes with F-actin and formats F-actin-enriched protrusive structures to promote gastric cancer cells migration and invasion 56. Another substrate of PAK1, Integrin-linked kinase (ILK), can be phosphorylated by PAK1 at threonine 173 and serine 246 to promote breast cancer cell motility and proliferation 57. After cancer cells penetrate blood vessels and enter the circulatory system, the cells are relatively fragile. They may have to adapt to different living environments after leaving the original suitable living circumstances. Once entering the circulatory system, it is easier for a cancer cell to be recognized and killed by the immune system, suffering from a variety of cellular pressures 58. Generally, there are relatively few cancer cells that can survive in the circulatory system. Fortunately, PAK1 can help the immune escape of tumor cells. Of note, miR-132, a tumor suppressor that can specifically impede hematogenous metastasis, can be regulated by PAK1 through PAK1-ATF2-miR132 cascade in gastric cancer. In short, PAK1 phosphorylates ATF2 at serine 62 to inhibit its transcription factor activity, and miR-132 happens to be one of its transcription activation objects 59. Finally, in the process of extravasation of cancer cells to the secondary area and to new tissues and organs, chronic inflammation induced by PAK1 promotes cancer cell survival in the new environment through the remodeling of the extracellular matrix. The inhibitory effect of PAK1 on apoptosis is also indispensable during the entire metastasis process 60, and the inhibition of apoptosis also ensures the survival of cancer cells to the greatest extent. Although cancer cells may face a bleak situation during metastasis, when they survive in new tissues, PAK1 can promote their adaptation to the new environment, rendering them dormant or enabling them to further breed.

### PAK1 in cancer immunity and metabolism

To survive, cancer cells must respond intelligently to the body's immune system and various metabolic pressures. During the survival and expansion of cancer cells, the immune system can recognize and eliminate some of the cancer cells, especially during the metastasis in the circulatory system. Interestingly, PAK1 can enable the immune evasion to adapt to different microenvironments by regulating cancer cells' metabolisms, such as switching to oxidative phosphorylation in an aerobic environment, or switching to fatty acid oxidation in a high-lipid environment. PAK1 can also induce the activation of NF-κB-mediated inflammatory response, thereby making cancer cells partly immune to escape 61. Recently, a study found in pancreatic ductal adenocarcinoma (PDA), PAK1 knockout improved the number of CD4^+^ and CD8^+^ T cells and inhibited the activation of pancreatic stellate cells (PSCs) 62. Furthermore, PAK1 is also involved in the elongation of activated T-cells 63. As we all know, hepatitis can cause liver cancer, while interestingly; PAK1 was confirmed to help HCV RNA replication through PI3K and ERK activation 64. In addition, the metabolic pattern of cancer cells is often very different from normal cells. Most solid tumors are more dependent on glycolysis for energy supply, and some brain tumors or blood cancers are more inclined to oxidative phosphorylation addiction. Importantly, PAK1 plays a charming role in affecting cancer cell metabolism. In cervical cancer and colorectal cancer, PAK1 can promote HIF1-α activation to adapt to oxygen-deficient conditions 46. Moreover, PAK1 can regulate glucose metabolism in cancer. Phosphoglucomutase 1 (PGM), which is a key glucose utilization regulator, was identified to be a substrate of PAK1. PAK1 phosphorylates PGM at threonine 466 to enhance its enzymatic activity. The activation of PGM further helps cancer cells glucose and energy homeostasis 65. Another study found that hyperglycaemic will lead to the activation of CDC42-PAK1 signaling, which may increase the oncogenic of breast cancer cells 66. In conclusion, the effects of PAK1 on immunity and metabolism can regulate the progression of cancer in a variety of situations.

### PAK1 in cancer drug resistance

Currently the biggest challenge in cancer therapy is cancer drug resistance. The constant emergence of resistance-related mutations, activation of compensatory pathways, and influence of other unknown factors have resulted in the situation that many drugs with good initial effects quickly lose their therapeutic effect. In addition, due to the heterogeneity of tumors, many anti-tumor treatments have artificially screened a group of super tumor cells that have evolved entirely and are not responding 67. Various factors accumulation makes cancer treatment outcomes often less than ideal. PAK1 has played a vital role in the drug resistance process in multiple tumors, and synergistic inhibition of PAK1 always benefits the tumor treatment (**Figure 2B**). In EGFR-mutated lung cancer, inhibitors of EGFR showed limited effectiveness because of specific site mutations. Since EGFR and PAK1 can both activate the Ras-MAPK and PI3K-AKT signaling pathways, inhibition of PAK1 synergistically enhances the inhibitory effect on these pathways. This is why PAK1 inhibitors can significantly enhance the therapeutic effect of EGFR-TKI inhibitors in EGFR-TKI-resistant non-small cell lung cancer and lung adenocarcinoma 68, 69. Furthermore, in chronic myeloid leukemia (CML), PAK1 inhibition displayed synergistic effect with TKIs 70. In pancreatic ductal adenocarcinoma, PAK1 activation triggers the Wnt/β-catenin signaling cascade that is involved in resistance to **Gemcitabine**. Therefore, it is not surprising that PAK1 inhibition can enhance the response of resistant and non-resistant cells to **Gemcitabine** 71. Similarly, the inhibition PAK1-regulated Wnt/β-catenin pathway also enhanced the sensitivity of cancer cells to **Cisplatin** in non-small cell lung cancer 72. In addition, PAK1 activation can enhance a cancer cell's resistance to BRAF inhibitors, which is mainly due to the activation of the AKT pathway. And the inhibition of PAK1 can suppress not only the activation of MEK-ERK but also the conduction of AKT signaling pathway 73. In **Tamoxifen**-resistant breast cancer cells, the inhibition of PAK1 restores the sensitivity of cancer cells to **tamoxifen** to improve the therapeutic effect 74. In BRAF-mutant melanomas, the activation of PAK1 is contribute to the acquired drug resistance to BRAFi and combined BRAFi and MEKi therapies 75. In renal epithelial cell carcinoma, PAK1 plays an important role in the activation of the NF-κB/IL-6 pathway, which involves **Sunitinib** resistance 61. PAK1 is also involved in the resistance of pancreatic cancer cells to MET inhibitors, and PAK1 inhibitor attenuated tumor growth and metastasis *in vivo* 76. Similar effects have been observed in lymphatics that are resistant to PI3K/mTOR inhibitors 77. As a core regulator of cell proliferation and metastasis networks, PAK1 plays a prominent role in cancer drug resistance.

Overall, PAK1 plays an important role in cancer progression, from the formation of cancer to the enhancement of the cancer cells' motor capacity, their infiltration of the circulatory system, and eventual metastasis to new tissues for survival and even cancer's drug response. Hence, the PAK1 has been identified as an attractive target for cancer treatment. Also, it is important to understand the regulation of PAK1 and the mechanism of regulation involved in PAK1. Next, we will further discuss the specific mechanism by which PAK1 participates in the course of the life of cancer cells.

## Endogenous upstream regulators of PAK1

**Figure 3:**
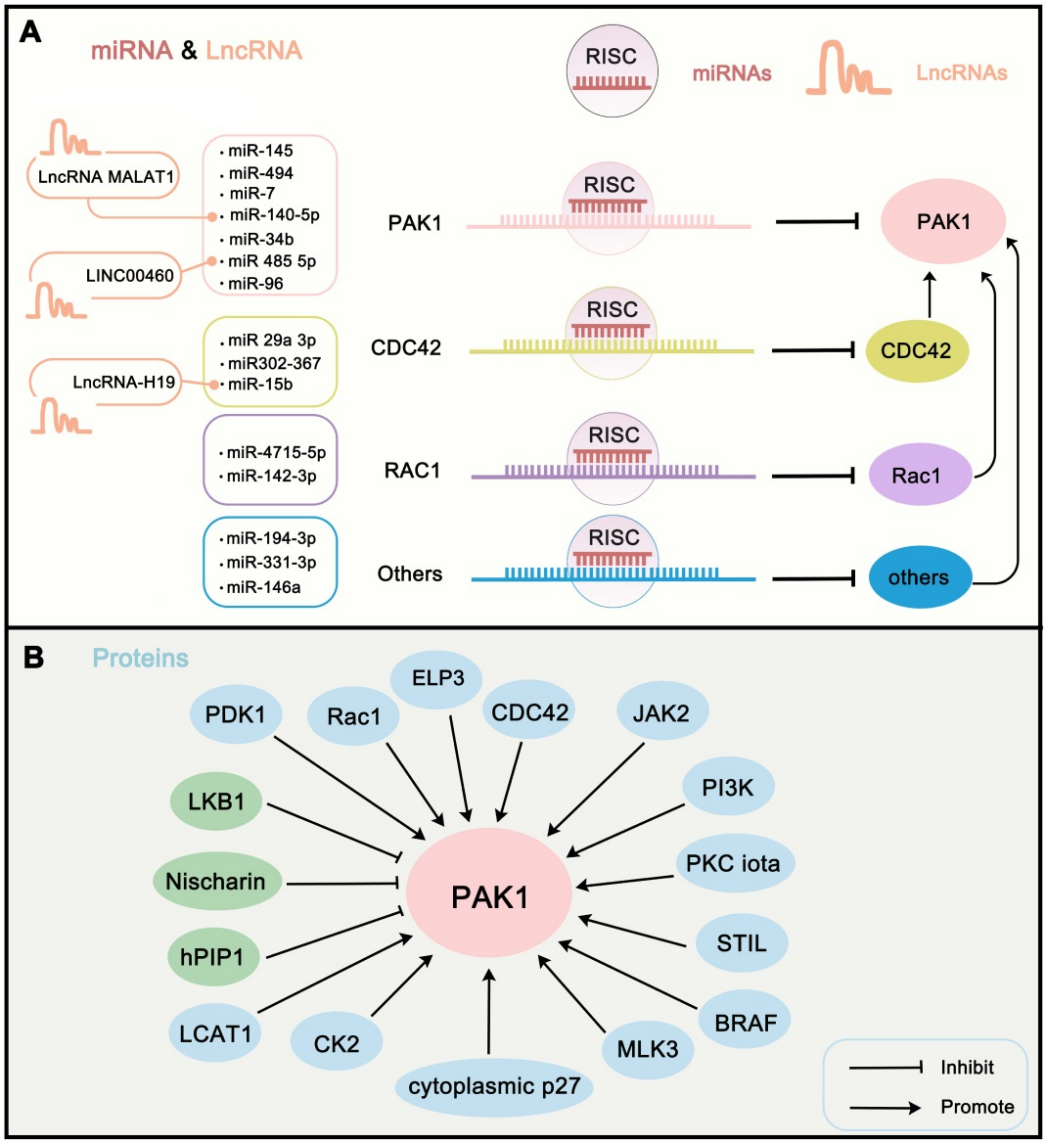
**Endogenous upstream regulators of PAK1.** (**A**) miRNAs and LncRNAs that involve in regulating the expression of PAK1. Of them, miRNAs target PAK1 or PAK-related proteins to regulate PAK1 expression. Moreover, LncRNAs regulate PAK1 activity via target PAK1-related miRNAs. (**B**) The protein interaction is another way to regulate PAK1 activity. The proteins can regulate PAK1 activity mainly through phosphorylation, acetylation, transcriptional regulation, and PPI network. The green proteins in the figure inhibit PAK1 activity and the blue proteins activate PAK1 activity.

To further understand the regulation mechanism of PAK1 in cells, we first summarize the endogenous upstream PAK1 regulators. Here, we review the regulation of PAK1 activity in terms of transcriptional regulation, post-transcriptional modification, and protein-protein interaction network. Transcriptional regulation of PAK1 activity is mainly mediated by microRNAs (miRNAs), which directly or indirectly recognize the mRNA of PAK1 and guide the silencing complex to degrade the PAK1 mRNA or to suppress the translation of PAK1 78. The regulation of miRNAs on target genes is also a hot spot in oncology. Studies have revealed that miR-485-5p 79, miR-140-5p 80, miR-96 81, miR-7 82, miR-494 83, miR-34b 84, and miR-145 85 can directly target and degrade the mRNA of PAK1 in order to block the translation of PAK1. In addition, PAK1 can be directly activated by interaction with Rho-related GTPases CDC42 and RAC1 86. However, some miRNAs targeting these activators can also regulate the activity of PAK1, such as miR-142-3p and miR-4715-5p targeting RAC1 87, 88, and miR302-367, miR‑29a‑3p, and miR-15b targeting CDC42 48, 89, 90. Other miRNAs perform other functions. For instance, miR-146a can target VEGF to inhibit VEGF/CDC42/PAK1 signaling 91; miR-331-3p directly targets ErbB2 and VAV2 and inhibits PAK1 activity through the ErbB2/VAV2/Rac1/PAK1 pathway 92; and miR-194-3p inhibits PAK1 activity through the PI3K/AKT/CDC42/PAK1 pathway 93. Certain long non-coding RNAs can target miRNAs to affect PAK1 activity. For instance, LncRNA-H19 suppresses miR-15b expression to activate the CDC42-PAK1 pathway 90. LINC00460 promotes tumor progression through sponging miR-485-5p and up-regulating PAK1 79, and MALAT1 interacts with miR-140-5p to abolish the inhibition of PAK1 80 (**Figure 3A**). In addition, transcriptional modification by transcription factor is another major regulation of PAK1. nonclustered H2.0-like homeobox (HLX) is a homeobox domain-containing transcription factors that can regulate PAK1 transcription 13. The post-transcriptional modification of PAK1 is mainly mediated by phosphorylation and acetylation. Except for RAC1 and CDC42, several other proteins can regulate PAK1 activity through phosphorylation. LKB1 can suppress PAK1 activity through phosphorylation of Thr109 94; MLK3 directly activates PAK1 kinase activity via phosphorylating PAK1 on Ser133 and Ser204 sites 95; and Her2, cytoplasmic p27, bFGF, CK2, JAK2, and PDK1 all can phosphorylate PAK1 to activate its activity 2, 96-100. In addition, elongator acetyltransferase complex subunit 3 (ELP3) can acetylate PAK1 at K420 to inhibit the dimerization of PAK1, which further promotes its activity 101. Of note, the protein-protein network is another way to regulate PAK1 activity. Certain proteins can indirectly affect PAK1 activity via RAC1 and CDC42 regulation, such as PKC iota 102, RIT1 103, NCK1 104, and P-REX1 105. Other tumor promoters, such as PI3K and CKIP-1, both regulate PAK1 activity via the protein-protein network 98. Some representative examples are shown in (**Table 1**) (**Figure 3B**). Through the summary of these endogenous upstream regulators of PAK1, in addition to an understanding of the internal regulation mechanism of PAK1, we also believe that, through the intervention of these factors, such as the development of ncRNA drugs for PAK1, the destruction of the PAK1-related PPI network might be beneficial for coming up with new strategies for cancer treatment.

## PAK1 regulated cancer-related pathways

PAK1 participates in multiple signaling pathways of cancer development through regulating various substrates. Here, we mainly review the specific mechanisms of PAK1 in Wnt/β-Catenin, EGFR/HER2/MAPK, NF-κB and apoptosis, cell cycle regulation, autophagy, EMT, metastasis pathways and the crosstalk between PAK1 and some other signaling.

### PAK1 in Wnt/β-Catenin pathway

**Figure 4:**
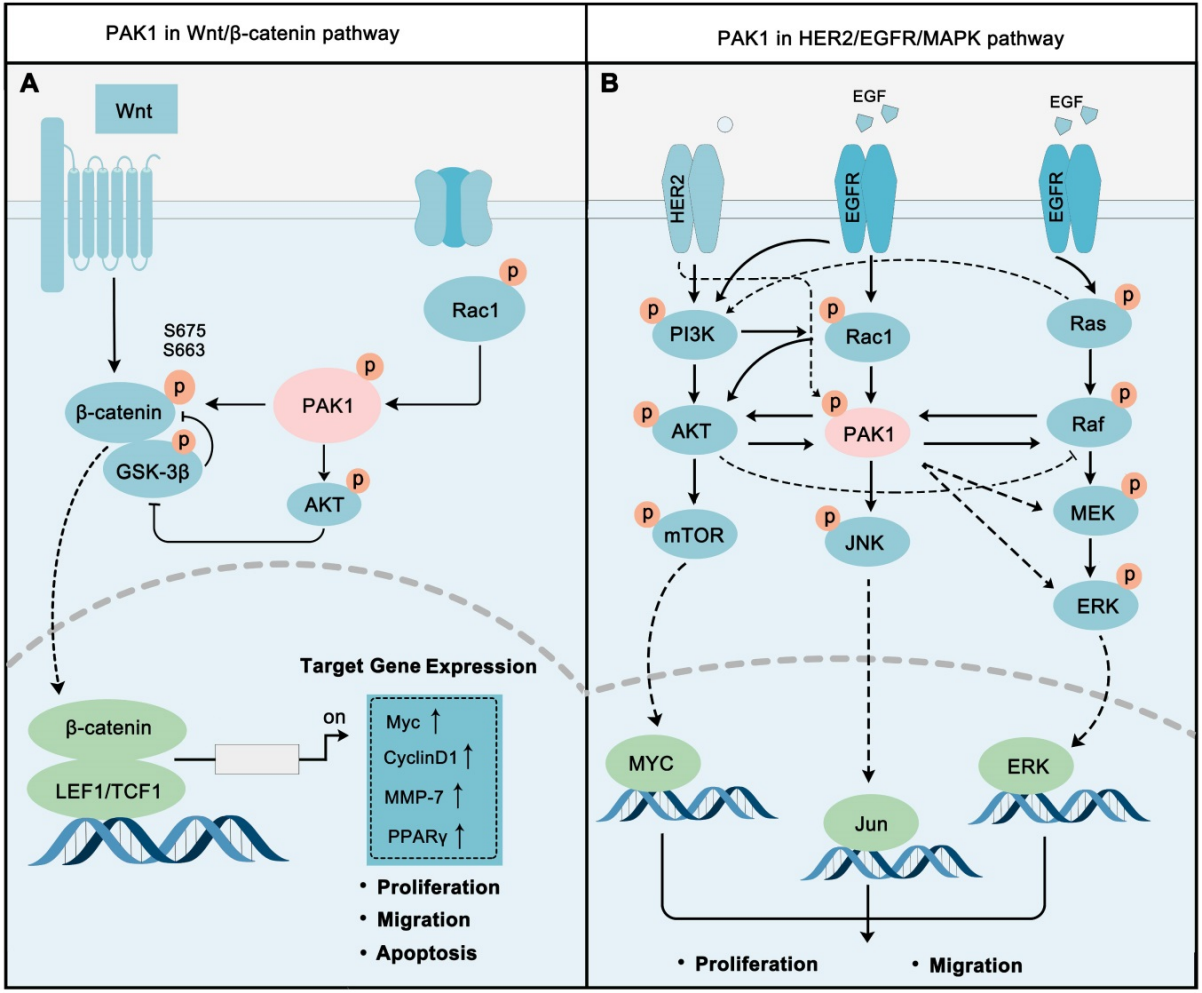
** PAK1 is a key molecular determinant in major cell proliferation pathways.** (**A**) PAK1 promotes Wnt / β-catenin signaling via activating β-catenin. The activation of β-catenin by PAK1 is mainly through direct phosphorylation and PAK1-AKT-GSK-3β pathway. After activation, β-catenin translocates to the nucleus and activates the transcription of a series of oncogenes, such as *MYC* and *MMPs*. (**B**) PAK1 is at the core of EGFR/HER2/MAPK pathways, and PAK1 precisely regulates EGFR/HER2/MAPK network to regulate cancer cell.

The Wnt signaling cascade is a primary regulator of human/animal growth and development, Wnt/β-Catenin pathway is one of most typical Wnt signaling pathways. The occurrence and development of multiple tumors are closely related to the abnormal activation of the Wnt/β-Catenin pathway 118-119. Wnts are basically growth stimulatory factors that promote cell proliferation, while β-Catenin is the key switch to regulate this signal 120. PAK1 plays a vital role in regulating this pathway (**Figure 4A**). On the other-hand, PAK1 activation leads to the phosphorylation of S675 and S663 of β-catenin to stabilize β-catenin, and then to activate the transcription of target genes, such as *MYC*, *CyclinD1*, *MMP,* and *PPARγ,* to promote cancer cell proliferation and migration 117. Also, PAK1 inhibits GSK3 activity via AKT1 activation to eliminate the inhibitory effect on β-catenin 121, 122. In fact, PAK1 and Wnt/β-Catenin do contribute to cancer development. For instance, PAK1 activation implicated in Wnt/β-Catenin drives early phases of oncogenesis and cancer growth in colon cancer 123. In non-small cell lung cancer, PAK1-β-Catenin-regulated cancer cell stemness contributes to chemoresistance 124. Activated PAK1 fine-tuning the Wnt/β-Catenin pathways contribute to regulating cancer proliferation and metastasis. The cooperative inhibition of PAK1 and Wnt/β-Catenin is a new strategy for cancer therapy.

### PAK1 in EGFR/HER2/MAPK pathways

The ERBB family of receptor tyrosine kinases are essential for tumorigenesis, epidermal growth factor receptor (EGFR), and ERBB2 (HER2) are prestigious. ERBB family receptors can activate multiple downstream oncogenic pathways, including PI3K-AKT and MAPK pathways, to regulate cell proliferation, migration, differentiation, apoptosis, and motility 125. EGFR and HER2 in cancer are often characterized by inappropriate activation, including point mutation, overexpression, partial deletions, and autocrine ligand-receptor stimulation 126, and their roles in cancer are best-defined 127. The activation of ERBB pathways can recruit and activate a series of downstream proteins involved in different signaling transduction by phosphorylation 128. In this section, we mainly focus on the role of PAK1 in the EGFR/HER2/MAPK network.

PAK1 is involved in the regulation of HER2-PI3K-AKT-mTOR pathway (**Figure 4B**). Studies have proven that PI3K activates PAK1 via phosphorylation of its upstream regulator Rac 129, and Rac can activate AKT. AKT and PAK1 can directly stimulate each other's activity 130. The crosstalk between the PI3K-AKT and MAPK-ERK signaling pathways has always been a hot spot in cancer research, and it is a significant contributor to cancer drug resistance. The combination of these two pathways allows cancer cells to survive in a variety of anticancer drugs. Specifically, Ras can directly activate PI3K, while AKT and Raf can regulate each other to switch cancer cell proliferation or cell cycle progression 131, 132. PAK1 also made itself a contribution in this network by activating Raf, MEK, and ERK, while Raf in turn activates PAK1 133, 134. PAK1 also contributes to cancer progression through inducing JNK activation 135.

Increasingly, studies have revealed the important role of PAK1-regulated EGFR/HER2/MAPK pathways in cancer. Inhibition PAK1 could block the Akt/mTOR signaling pathway to benefit breast cancer therapy 136. Reducing PAK1 activity will diminish the proliferation of malignant peripheral nerve sheath tumors (MPNSTs) through inhibiting the Raf/Mek/Erk pathway 137. PAK1 occupies a relatively central position in the EGFR/HER2/MAPK pathway network. The co-inhibition of PAK1 and other core proteins in the network, such as AKT and MEK, may also become a new strategy for cancer treatment.

### PAK1 in NF-κB and apoptosis pathways

**Figure 5:**
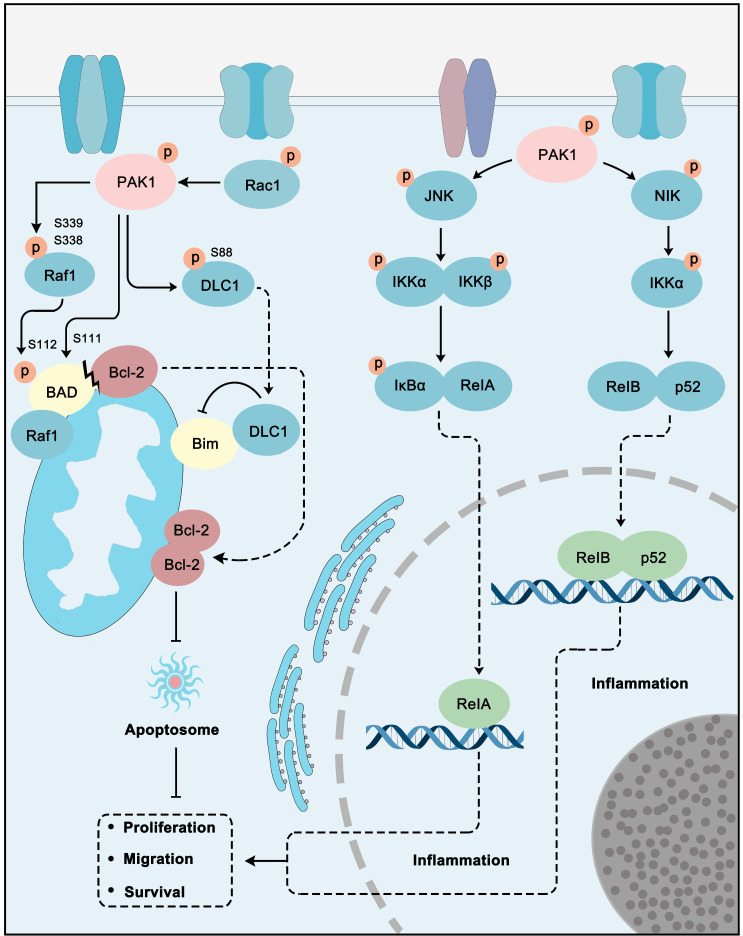
** PAK1 plays a central role in apoptosis and NF-κB pathways.** PAK1 can inhibit apoptosis through Raf1 and DLC1 regulated Bim inhibition and Bcl-2 activation. In addition, PAK1 stimulate JNK-IκB-RelA and NIK-IKKα-RelB/p52 pathways to promote inflammation to promote cancer cell proliferation, migration and survival.

NF-kB signaling directly or indirectly controls cancer cells inflammation, proliferation, EMT, survival, angiogenesis, invasion, and metastasis. Also it controls cancer stem cell formation, stress responses, cell metabolism, immunosuppression, and further therapeutic resistance. NF-κB activation is often observed in malignant cells and tumor microenvironments 138. NF-κB also implicates apoptosis regulation in cancer cells 139. Apoptosis is an evolutionarily conserved pathway of cell death and plays an essential role in proper development and maintaining homeostasis. Apoptosis is mainly controlled by BCL-2 family proteins containing pro-apoptotic and pro-survival members, as well as determines the cell's life and death according to the environment and pressure that the cell encountered. Inducing cancer cell apoptosis has long been the main strategy for cancer treatment 140. PAK1 also performs roles in the regulation of apoptosis and NF-κB pathway for cancer development (**Figure 5**). For example, the growth hormone-releasing hormone receptor (GHRH-R) can promote human gastric cancer via PAK1-NF-κB signaling 141. First, PAK1 inhibits apoptosis via suppressing the pro-apoptotic protein (BAD, Bim) and activating the pro-survival protein (Bcl-2). Functionally, PAK1 phosphorylates Raf-1 at Ser-338 and Ser-339 to promote its translocation to mitochondria for further phosphorylation of BAD at Ser-112. Mitochondria Raf-1 disrupts the formation of Bcl-2-BAD complex and enhances the anti-apoptosis ability of Bcl-2 142. Pak1 can also directly phosphorylate BAD at Ser111 143. PAK1 can also phosphorylate a dynein light chain 1 (DLC1) to resist apoptosis via interacting with pro-apoptotic protein Bim 144. PAK1 promotes the NF-κB pathway through stimulating JNK and NF-κB interacting kinase (NIK). JNK can phosphorylate IKKα/β to accelerate IκB phosphorylation. RelA is then released from the IκB complex and is activated. NIK activates IKKα, which in turn activates the RelB-p52 complex by phosphorylation. The activated RelA and RelB-p52 translocate to the nucleus to induce the stimulation of target genes involved in cancer proliferation, migration, and survival 44. The conclusion is that PAK1 inhibition could be a favorable strategy for NF-κB-dependent and apoptosis-resistant cancer treatment.

### PAK1 in cell cycle pathways

**Figure 6:**
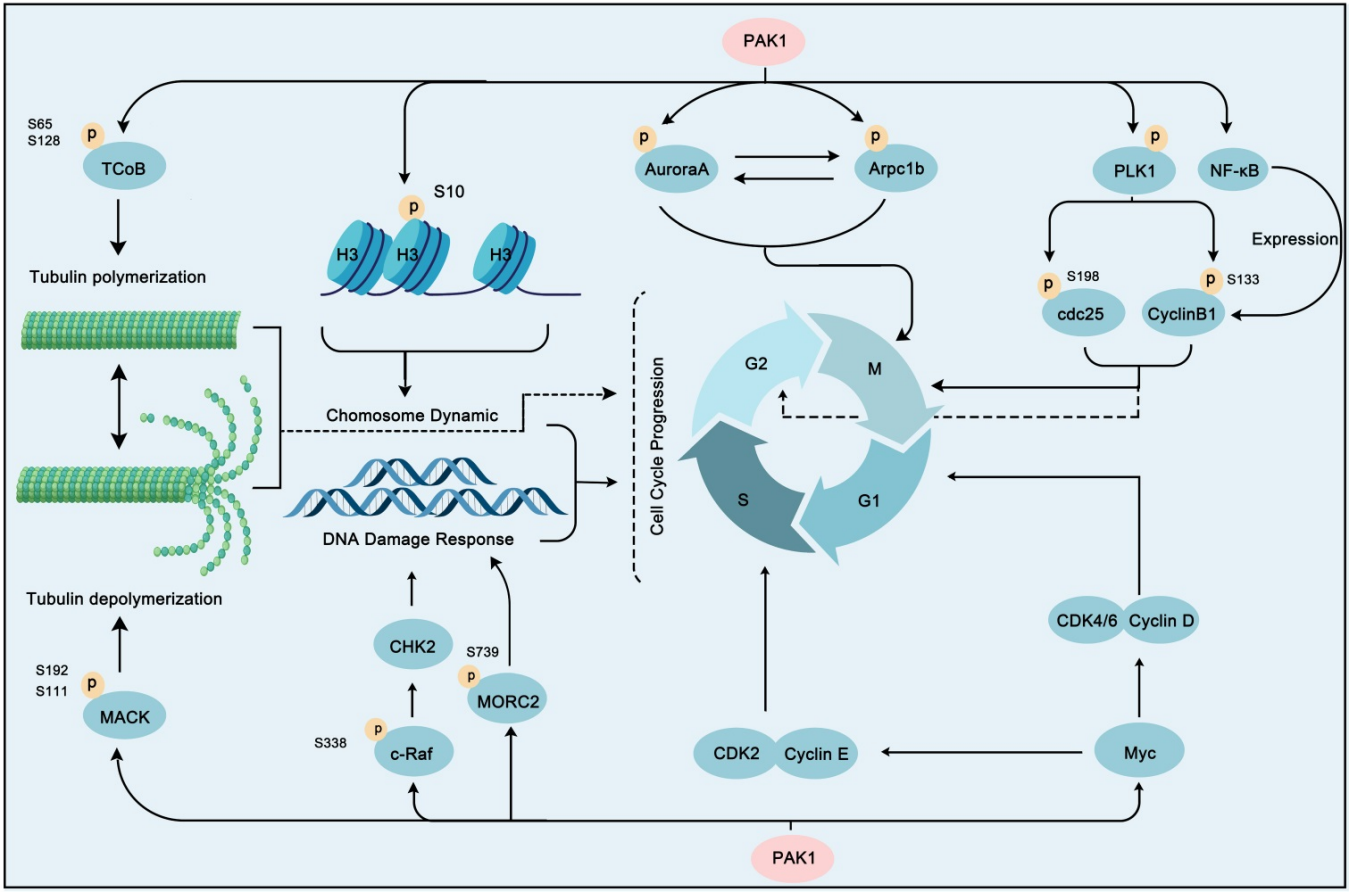
** PAK1 contributes a lot in cell cycle progression.** PAK1 triggers cell cycle progression through phosphorylation of some substrate proteins, including AuroraA, Arpc1b, PLK1, histone H3, MORC2, NF-κB and c-Raf. In addition, PAK1 activates Myc to stimulate the activity of CDK2/CyclinE and CDK4/6/CyclinD to promote cell cycle profression. Moreover, PAK1 can regulate tubulin polymerization and polymerization via TCoB and MACK to play a role in cell cycle regulation.

A common phenomenon in cancer is abnormal expression or regulation of cell cycle proteins, which leads to uncontrolled cell proliferation. Cell proliferation depends on the regulation of the cell cycle progress, which is generally divided into four phases: G0/G1, S, G2, and M phases. This process is directly regulated by cyclin-dependent kinases (CDKs), checkpoint kinases, Aurora kinases, and Polo-like kinases (PLKs) 145. Intriguingly, these proteins are directly or indirectly regulated by PAK1 (**Figure 6**). Aurora A, an important centrosomal kinase, can be activated by PAK1 by phosphorylation at Thr288 and Ser342, and then stimulates mitosis 146. Therefore, PAK1 and Aurora A dual-inhibition could benefit breast cancer treatment 147. In addition, PAK1 phosphorylation of the actin-related protein 2/3 complex subunit 1B (Arpc1b) can regulate actin networks, which contributes to cell motility 54. Arpc1b and Aurora A are both activator and substrate of each other 148. The PAK1-induced activation of Arpc1b and Aurora A initiates a series of subsequent caspase cascades that contribute to mitosis. Microtubules dynamics regulation is also the main way of PAK1 to regulate mitosis. Microtubules have the dynamic characteristics of polymerization and depolymerization, which play an important role in the movement of chromosomes during mitosis. Tubulin cofactor B (TCoB), which exhibits overexpression and hyperphosphorylation in breast cancer can be directly bound and phosphorylated on serine 65 and 128 by PAK1, and then interact with tubulin to regulate the polymerization of new microtubules and mitosis 55. Interestingly, mitotic centromere-associated kinesin (MCAK), another substrate of PAK1 can regulate microtubule depolymerization when phosphorylated by PAK1 on serine 192 and 111 149. Furthermore, the precise regulation of these two substrates by PAK1 constitutes the epitome of regulating mitosis by regulating microtubule dynamics. Strikingly, PLK1 activation is another strategy that PAK1 regulates mitosis. PLK1 is an important cell cycle regulator that can enable mitotic entry by phosphorylating cdc25 and cyclinB1 150, 151. Interestingly, PAK1 can also regulate cyclinB1 activity via transcription and expression enhancing through NF-KB activation in gastric cancer 152. Moreover, the oncogenic transcription factor *MYC,* another crucial downstream effector of PAK1, can regulate several cyclin related kinases (CDK4/6, cyclin D, CDK2, and cyclin E) to promote cell cycle progression 153. The phosphorylation of Raf on Serine 338 by PAK1 promotes its kinase activity to activate cell cycle checkpoint kinase 2 (CHK2) to regulate the DNA damage response, which contributes to cancer cell survival 15. In addition, PAK1 can affect the cell cycle by regulating the dynamic changes of chromosomes. For one case, PAK1 phosphorylates histone H3 to regulate chromosome dynamic for further cell cycle regulation in breast cancer cells 154. For another case, PAK1 phosphorylated MORC2 at Serine 739 when cells encounter DNA damage, and the phosphorylation of MORC2 then facilitates chromatin remodeling 42. Considering the vital role of PAK1 in the cell cycle, several studies have revealed that dual targeting PAK1 inhibition and cell cycle destruction would reap significant benefits in cancer therapy 155, 156.

### PAK1 in autophagy pathways

**Figure 7:**
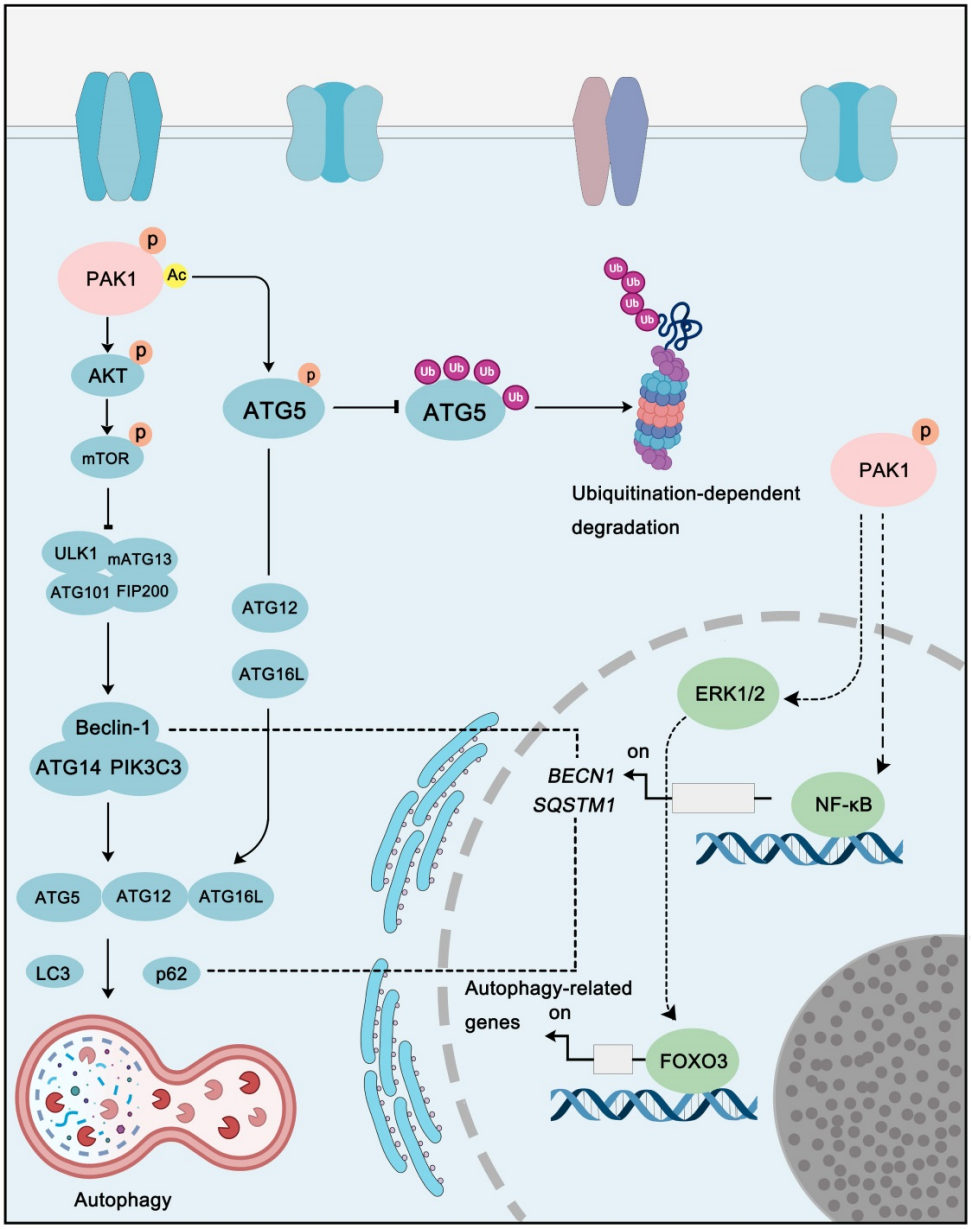
** PAK1 in autophagy pathways.** PAK1 regulates autophagy via direct or indirect regulation. Acetylation of PAK1 could phosphorylate ATG5 to inhibit its ubiquitination-dependent degradation to promote autophagy. In addition, PAK1-AKT-mTOR signaling is another key pathway that involved in PAK1-regulated autophagy. Moreover, PAK1-mediated autophagy-related genes expression is also contribute to autophagy regulation.

With Yoshinori Ohsumi winning the Nobel Prize in Physiology or Medicine for his research on autophagy, the role of autophagy in cancer has gradually become a hot spot in cancer diagnosis and therapy, and for its dual-role, study on autophagy in cancer is even more fascinating. Interestingly, PAK1 also plays a role in the regulation of autophagy via direct or indirect mechanism. For instance, PAK1 can phosphorylate at ATG5 at the Thr101 to prevent its ubiquitination-dependent degradation. In addition, the phosphorylation of ATG5 by PAK1 also promotes the formation of ATG5-ATG12-ATG16L, which is one of the core complexes of autophagy with E3 ligase activity, and PAK1-ATG5 induced autophagy promotes glioblastoma (GBM) growth 101. As to indirectly regulation, PAK1 can activate AKT-mTOR pathway, and interestingly, mTOR is not only a key cell growth regulator, but also an important autophagy-related protein. In breast cancer, the inhibition of PAK1 by **Ivermectin** could inhibit AKT activity thus decreasing mTOR activity to induce cytostatic autophagy 157. In addition to these examples, the regulation of PAK1 on other pathways, such as MAPKs and NF-κB, can indirectly regulate autophagy as well (**Figure 7**). Therefore, considering the relationship between PAK1 and autophagy, the development of a therapeutic regimen targeting PAK1-autophagy might be a potential strategy for cancer treatment.

### PAK1 in EMT and metastasis pathways

**Figure 8:**
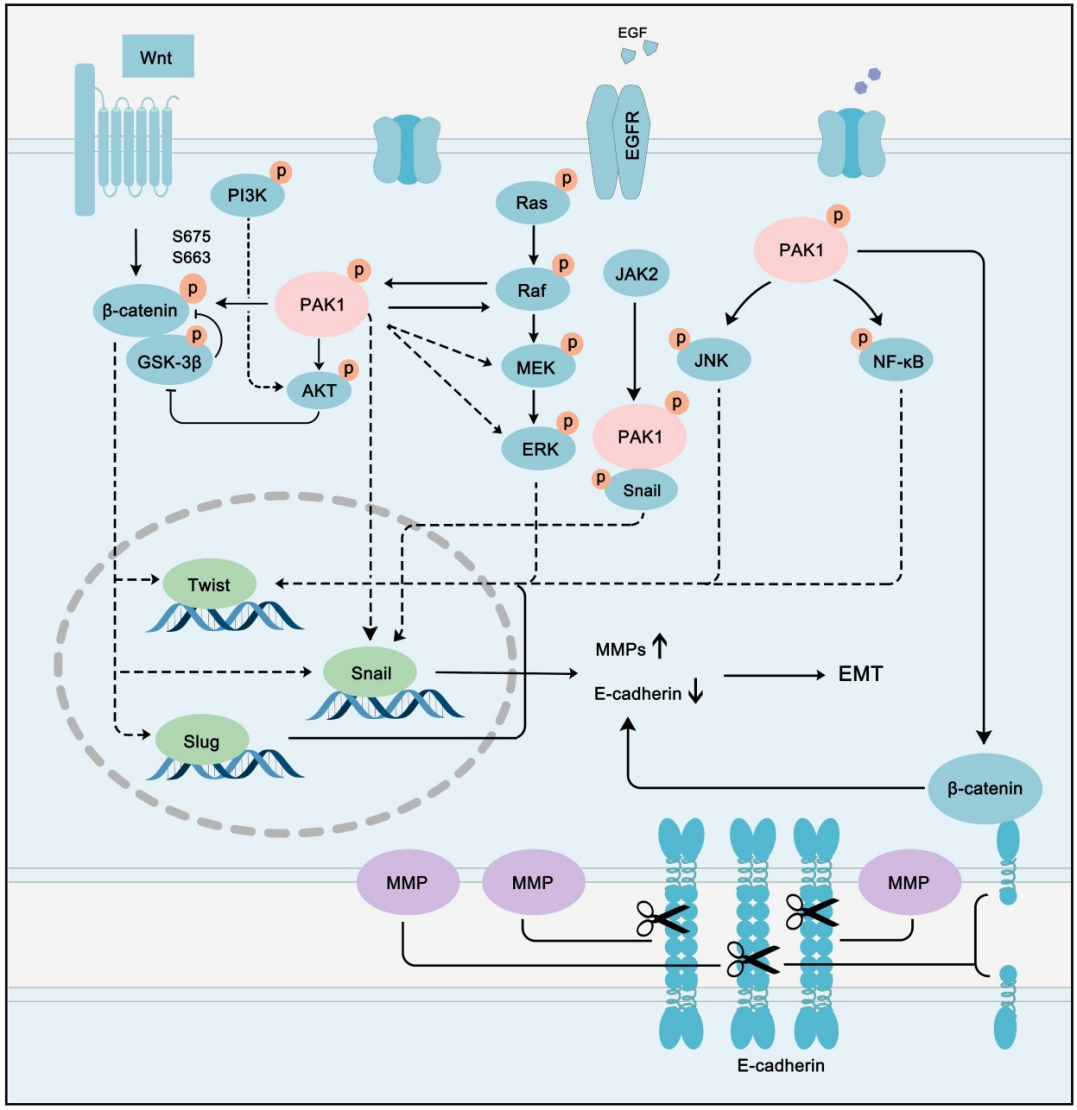
** PAK1 fine-tuning the PPI network to accelerate cancer EMT and metastasis.** PAK1 can activate Wnt/β-catenin, PI3K-AKT, Ras-MAPK, JNK and NF-κB pathways to promote the transcription of twist, snail and slug. These EMT-promoting transcription factors promote the expression of MMPs, while inhibit the expression of epithelial cell specific protein, such as E-cadherin to promote cancer EMT and metastasis.

The role of PAK1 in EMT and metastasis has been a hot topic in the field of cancer. Numerous studies have confirmed that the PAK1-regulated pathway network contributes to EMT and metastasis in multiple types of cancer. In colon cancer, PAK1-LIMK1-Cofilins signaling is a driving force of EMT 158. The PAK1/β-catenin pathway also plays an essential role in ovarian cancer. 159. Snail up-regulation and β-catenin activation induced by PAK1 are the main processes of cancer metastasis in hepatocellular carcinoma 160. In parallel to hepatocellular carcinoma, PAK1 can promote Snail transcription by directly binding to Snail in lung cancer cells 161. PAK1 promotes cancer EMT and metastasis through a network of multiple pathways. A hallmark of EMT is the loss of epithelial cell integrity resulting from degradation of the adhesive connections that maintain contact between epithelial cells. This degradation is mainly accomplished by MMPs driven by EMT-promoting transcription factors, such as twist, snail, and slug 162. Intriguingly, these EMT-promoting transcription factors can all be activated by PAK1. PAK1 can activate Wnt/β-catenin, PI3K-AKT, Ras-MAPK, JNK, and NF-κB pathways, which can promote the transcription of twist, snail, and slug. These EMT-promoting transcription factors promote the expression of MMPs, while inhibiting the expression of epithelial cell-specific proteins, such as E-cadherin 163 (**Figure 8**). Cancer cells can further metastasize and deteriorate under the programming of a signal network regulated by PAK1. Although the multi-faceted promotion of cancer by PAK1 is disturbing, it also provides an opportunity for the treatment of most PAK1-dependent cancers.

### Cross-talk between PAK1 and other signaling pathways

**Figure 9:**
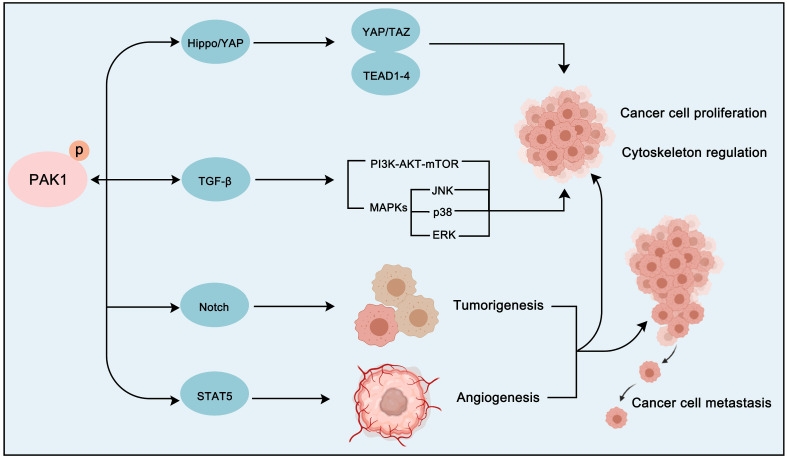
** The crosstalk between PAK1 and other cancer-related pathways.** PAK1 can also regulate the proliferation and cytoskeletal movement of cancer cells via crosstalk with the Hippo-YAP signaling pathway and the TGF-β signaling pathway. In addition, PAK1 can promote tumorigenesis and angiogenesis through Notch and STAT5 signaling pathways activation.

In addition to the classical cancer pathways that PAK1 mainly participates in, the crosstalk between PAK1 and other pathways is also crucial for the development of cancer (**Figure 9**). For instance, transforming growth factor-beta (TGFβ) pathway, which is an important regulatory pathway in cellular life, is also a key pathway in cancer. And interestingly, TGFβ pathway plays a two-sided role in cancer 164. It is worth noting that the crosstalk between PAK1 and TGFβ pathway is also important in cancer proliferation, angiogenesis, EMT and metastasis regulation. In prostate cancer, PAK1 was found to inhibit TGFβ expression to promote cancer cell growth and migration. Intriguingly, the overexpression of TGFβ in turn enhances the activation of PAK1 to promote EMT 165, 166. In non-small-cell lung cancer (NSCLC), the up-regulation of transforming growth factor-β receptor type 1 (TGFBR1) can also stimulate the activation of PAK1 to promote tumorigenesis 167. Except for TGFβ signaling, Notch, Hippo/YAP and STAT5 signaling pathways are also involved in PAK1-regulated cancer progression. Importantly, both PAK1 and Notch often exhibit dysregulation in tumors. SHARP, a co-repressor of Notch signaling, was found to be a substrate of PAK1. And PAK1 phosphorylated SHARP at Ser3486 and Thr3568 to inhibit the activation of Notch target genes to regulate cancer destiny 168. As for Hippo/YAP cascade, the activation of FAK-ILK-PAK1 signaling will suppress the Hippo/YAP pathway to inhibit its tumor-suppressor role thus promoting the liver cancer cells cytoskeletal modulation 169. Remarkably, the crosstalk between PAK1 and STAT5 signaling is of great significance in the treatment and prognosis of leukemia. We all know that FLT3 and KIT oncogenic mutants are critical for the development of leukemia. A study found that these oncogenic mutants will activate FAK/Tiam1/Rac1/PAK1 signaling to further activate STAT5 and form a Rac1-PAK1-STAT5 complex to promote leukemogenesis via several oncogene activations 170. And the BCR-ABL fusion gene (BCR-ABL) is another oncogenic factor of leukemia which exhibits different subtypes with discrepant prognosis. A recent study discovered that the PAK1-STAT5 axis contributes to the different prognosis of BCR-ABL subtypes 171. Therefore, the crosstalk between PAK1 and other pathways is also not negligible for the treatment of cancer. We can develop a treatment strategy targeting PAK1 PPI network that is personalized according to the molecular characteristics of particular cancer.

## PAK1 inhibitors as a strategy for cancer therapy

Given the extensive cancer-related functions of PAK1, significant efforts have been made to develop PAK1 inhibitors from pharmaceutical and academic settings. According to the different binding modes in the kinase domain, these PAK1 inhibitors are classified into ATP-competitive inhibitors and allosteric inhibitors.

### ATP-competitive PAK inhibitors

#### Indolocarbazole-based inhibitors

K-252a, an indolocarbazole alkaloid isolated from *Nocardiopsis sp.*, was the first small molecule to be reported as a PAK1 inhibitor with a Ki value of 2.4 nM, but it only showed weak antiproliferatory activity *in vitro* 172, 173. Based on the K-252a scaffold, several K-252a derivatives were designed and synthesized, including the noted KTD606 (a K252a dimer) and CEP1347, which could suppress the proliferation of v-Ha-RAS-transformed NIH 3T3 cells but not normal cells 174. Staurosporine, another natural product with an indolocarbazole scaffold, was discovered to be a potent ATP-competitive PAK1 inhibitor with a sub-nanomolar level affinity against a broad range of kinases, particularly against PAK1 with an IC_50_ value of 0.75 nM 175. Although the poor kinase selectivity of Staurosporine has limited its preclinical use, it is still a useful drug and a promising lead for optimization to obtain higher kinase selectivity PAK1 inhibitors. Λ-FL172 is a staurosporine-inspired octahedral ruthenium complex, which was documented as a potent PAK1 inhibitor with an IC_50_ of 130 nM. Λ-FL172 showed an improved kinase selectivity that resulted from the space effect caused by the occupation of the rigid and bulky ruthenium complexes against the ATP-binding pocket 176. Subsequently, the author further simplified the structure of Λ-FL172 by locating the metal atom at different positions within the active site to yield (R)-1, which possesses a higher affinity (IC_50_ of 83 nM), but less kinase selectivity 177.

### Aminopyrazole-based inhibitors

The aminopyrazole moiety is a common and vital kinase inhibitor scaffold, which functions as an ATP adenine mimetic agent with the capacity of initiating hydrogen bond interactions within the PAK kinase hinge region. In 2007, Pfizer disclosed numerous 5-substituted monocyclic aminopyrazoles as PAK inhibitors, which opened the gate to the development of PAK1 inhibitors based on the aminopyrimidine scaffold 178. In 2013, other new aminopyrazole-based PAK1 inhibitors for cancer treatment and hyper-proliferative disorders were reported by Genentech. Among these derivatives, II-11 displayed the strongest PAK1 inhibitory potency with a Ki value of 11 nM and a cellular IC_50_ of 43 nM 179. PF-3758309, a pan-PAK inhibitor with bicyclic aminopyrazole, was identified as a potent, orally available PAK1 inhibitor with a Ki value of 14 nM 180. Due to its prominent antitumor activity and pharmacokinetic profile *in vivo*, PF-3758309 has progressed to Phase I clinical trials as an anticancer agent in patients with advanced/metastatic solid tumors 181.

### 2-amino pyrido[2,3-d] pyrimidine-7(8H)-one-based inhibitors

The 2-amino pyrido[2,3-d]pyrimidine-7(8H)-one derivatives were first identified as PAK1 inhibitors by Afraxis in 2007 182. Subsequently, numerous derivatives were reported in a series of patents 183-184. These compounds were originally used for the treatment of various CNS disorders, with the scope of their use soon being expanded to oncology indications 185. FRAX597, a noted potent PAK1 inhibitor with an IC_50_ value of 7.7 nM, displayed a selectivity of PAK1 over PAK4 of > 130-fold, which presented a potent antiproliferatory capacity against NF2-deficient schwannoma cells and significant anti-tumor activity in an orthotopic model of NF2 186. FRAX486, a derivative of FRAX597, was originally designed to treat Fragile X syndrome and showed a capacity of inhibition of prostate stromal cell growth 187. Additionally, by employing an unorthodox Low‑pKa polar moiety, G555 was documented as a potent, selective PAK1 inhibitor with an IC_50_ value of 3.7 nM, making it a useful small-molecule probe for the elucidation of PAK1 unique biological functions 188.

### Aminopyrimidine-based inhibitors

Other important PAK1 inhibitors were designed based on the aminopyrimidine core. Xu et al. developed 2-arylamino-4-aryl-pyrimidines as potent PAK1 inhibitors, with compound **12** showing potent inhibitory activity against PAK1, but the cell activity was not evaluated 189. In order to improve the selectivity against PAK1, the 5-cyclopropyl-1H-pyrazole group was incorporated into the 4-site of aminopyrimidine core for targeting the ribose pocket to yield a series of potent, selective PAK1 inhibitors, including compounds **13**, **14,** and **15** 190-192. To mitigate the toxicity, a pyridone side chain analog G-9791 was discovered as a selective PAK1 inhibitor 193. Compound **17** was a potent PAK1 inhibitor discovered through a high-throughput virtual filtering strategy 194.

### Other ATP-competitive inhibitors

OSU-03012, a previously characterized PDK1 inhibitor derived from celecoxib, displayed PAK1 inhibitory activity with an IC_50_ value of 7.7 nM, which enabled motile NPA thyroid cancer cells to migrate 195. AK963, a simple urea derivative, presented a PAK1 inhibitory potency and suppressed the proliferation of gastric cancer cells by downregulation of the PAK1-NF-κB-cyclinB1 pathway 196. Recently we reported a novel PAK1 inhibitor, ZMF-10, which presented an IC_50_ value of 174 nM with good selectivity. ZMF-10 induced significant ER-Stress, suppressed migration via FOXO3 activation, JNK1/2, ERK1/2 and AKT signaling inhibition 197.

### Allosteric PAK1 inhibitors

IPA-3, the first reported PAK1 allosteric inhibitor, could covalently form a cysteine adduct with a surface-exposed cysteine residue in the N-terminal, regulatory portion of PAK1. It was reported that IPA-3 could induce cell death and affected cell adhesivity to fibronectin in human hematopoietic cells 198. Compounds 22 and 23, both dibenzodiazepine derivatives, were discovered through a structure-based optimization strategy. These compounds bond to the PAK1 allosteric site, confirmed by crystallography 199. Compound 23 is the most active allosteric inhibitor of PAK1 currently reported, which would contribute to investigation on the biological functions of the PAK1 kinase. Using an ELISA-based screening protocol, a series of naphtho(hydro)quinone-based small molecules that allosterically inhibit PAK activity was identified, and 2-Mc-1,4-NHQ showed the most potent inhibitory activity, with a Ki value of 1.98 μM, disrupting the Cdc42-PBD interactions 200.

Although many potent PAK1 inhibitors have been reported, including ATP competitive inhibitors and allosteric inhibitors, the development of PAK1 inhibitors still faces some challenges. First, the selectivity of these inhibitors needs to be further improved, especially for ATP competitive inhibitors. Additionally, the chemical structure diversity of these inhibitors has a large exploration space, and the druggability of most inhibitors needs to be further evaluated to ensure their clinical application. Therefore, more efforts should be devoted to the discovery of novel PAK1-targeted inhibitors.

## Conclusions

Recently PAK1 has attracted wide attention due to its important role in regulating the skeleton and the movement of cancer cells. Furthermore, PAK1 has come to be seen as a cancer hallmark which regulates cytoskeleton dynamics, proliferation, metabolism reprogramming, mitosis, invasion, metastasis, tumor microenvironment response, cancer cell immune escape and cancer drug resistance. Due its huge impact on tumor survival, PAK1 has also emerged as a promising target for antitumor drug development.

PAK1 participates in almost every stage of a cancer cell and in every indispensable signal pathway, including EGFR/HER2/MAPK, Wnt/β-catenin, JNK/c-jun, NF-κB, cell cycle, apoptosis, autophagy and others, such as TGF-β and STAT5 signaling. The mutation, transmission or amplification of relating signaling pathway genes can affect the fate of cancer cells. PAK1 acts as a hub to intelligently and accurately connect these signals, and through the most appropriate regulation, to meet the comfort, metabolic stability, immune escape, survival and further development of cancer cells under different survival conditions. It acts like a core railway station, always ensuring the normal transportation capacity of cancer cells.

As PAK1 is a well-characterized promoter of the progression of cancer and a criminal in cancer development, and PAK1 inhibition is a good target for many cancer treatments. Given that the PAKs display distinct as well as overlapping functions, the pan-PAK inhibitors will inevitably cause severe side effects, although their tumor-killing effect may be stronger. Therefore, PAK1 targeting-inhibitors are a better option for the precise treatment of cancers. However, due to the high homology of the kinase domain, the design of a highly selective PAK1 inhibitor remains a significant challenge. To solve this issue, various discovery strategies could be utilized including high-throughput screening (HTS), virtual screening (VS), fragment-based drug design (FBDD), and structure-based drug design (SBDD), as well as drug repurposing. Allosteric inhibitors targeting PAK1 display superiority in selectivity compared with the pan-kinase inhibitors. Additionally, proteolysis targeting chimera (PROTAC) is an emerging novel technology that takes advantage of a small molecule to control intracellular protein levels through recruiting target proteins to the ubiquitin/proteasome system for selective degradation. We predict that the PAK1 PROTAC degradation agent will be one of the preferred strategies to achieve selectivity. Other strategies include bivalent inhibitors and covalent inhibitors. Considering that PAK1 plays an important role in the network of cancer-dependent proteins, selective co-regulation of these proteins is also a good solution. And Dual inhibitors do have shown significant advantages in overcoming drug resistance, with the potential targets, including BRD4/PAK1, EGFR/PAK1, HSP90/PAK1, and CDKs/PAK1. Targeting PAK1 with small molecules holds promise as a viable therapeutic strategy for cancers. However, the road toward market approval remains a long one, and this effort will require interdisciplinary collaboration. The discovery of potent and specific PAK1 inhibitors with high isoform selectivity will fill roles in multiple applications as useful pharmacological probes for elucidating PAK1 biological functions and in the development of medications to benefit patients in the near future.

In addition, considering the role of PAK in tumor immune escape, the selective use of PAK1 inhibitors or modulators (such as siRNAs, miRNAs, LncRNAs or antibody drugs) in immunotherapy may also be a candidate for adjuvant cancer treatment. Moreover, the dual role of PAK1 in autophagy makes its regulators combined with autophagy-regulating drugs may solve the problem of drug resistance induced by protective autophagy in chemotherapy. Overall, PAK1 has become a hallmarker and therapeutic target for a variety of cancers. By exploring the complex regulatory molecular mechanism of PAK1 in cancer progression and the current situation of small molecule drug development, it might be helpful to develop better PAK1 anticancer drugs and novel PAK1-related cancer treatment strategies.

## Figures and Tables

**Table 1:** Endogenous upstream regulators of PAK1

Name	Classification	Action mode	Regulatory Mechanism	References
iR-142-3p	Tumor suppressor	Inhibition	miR-142-3p directly target RAC1 to inhibit RAC1/PAK1 pathway	87
miR-146a	Tumor suppressor	Inhibition	miR-146a directly target VEGF to inhibit VEGF/CDC42/PAK1 signaling	91
miR-485-5p	Tumor suppressor	Inhibition	miR-485-5p targets PAK1 and suppresses its expression	79
miR-331-3p	Tumor suppressor	Inhibition	miR‐331‐3p directly targets ErbB2 and VAV2 and inhibits PAK1 activity through the ErbB2/VAV2/Rac1/PAK1 pathway	92
miR-140-5p	Tumor suppressor	Inhibition	miR-140-5p directly targets PAK1 to inhibit its expression	80
miR-4715-5p	Tumor suppressor	Inhibition	miR-4715-5p inhibits the activity of Rac1 to inhibit PAK1	88
miR-96	Tumor suppressor	Inhibition	miR-96 targets PAK1 to inhibit its expression	81
miR-194-3p	Tumor suppressor	Inhibition	miR-194-3p inhibits PAK1 activity through PI3K/AKT/CDC42/PAK1 pathway	93
miR302-367	Tumor suppressor	Inhibition	miR302-367 inhibits PAK1 activity through CDC42/PAK1 Pathway	49
miR-7	Tumor suppressor	Inhibition	miR-7 targets PAK1 to inhibit its expression	82
miR‑29a‑3p	Tumor suppressor	Inhibition	miR‑29a‑3p inhibits PAK1 activity through CDC42/PAK1 Pathway	89
miR-494	Tumor suppressor	Inhibition	miR‑494 suppresses PAK1 expression	83
miR-34b	Tumor suppressor	Inhibition	miR‑34b suppresses PAK1 expression	84
miR-145	Tumor suppressor	Inhibition	miR‑145 suppresses PAK1 expression	85
miR-15b	Tumor suppressor	Inhibition	miR-15b interacts with CDC42 and inhibits CDC42-PAK1 pathway	90
Nudt21	Tumor suppressor	Inhibition	Nudt21 inhibits Pak1 expression through its 3'-UTR alternative polyadenylation	106
merlin	Tumor suppressor	Inhibition	merlin inhibits the activation of PAK1 through binding to the PBD of PAK1	107
LKB1	Tumor suppressor	Inhibition	LKB1 suppresses PAK1 by phosphorylation of Thr109.	94
Nischarin	Tumor suppressor	Inhibition	Nischarin inhibits PAK1 kinase activity via interaction with the C-terminal domain of PAK1	108
hPIP1	Tumor suppressor	Inhibition	hPIP1 blocks PAK1 autoactivation	109
LncRNA-H19	Tumor promoter	Activation	LncRNA-H19 suppresses miR-15b expression to active CDC42-PAK1 pathway	90
LINC00460	Tumor promoter	Activation	LINC00460 promoted tumor progression through sponging miR-485-5p and up-regulating PAK1	79
miR-130b	Tumor promoter	Activation	miR-130b inhibits ARHGAP1 expression to active CDC42-PAK1 pathway	110
LncRNA MALAT1	Tumor promoter	Activation	MALAT1 interacts with miR-140-5p and inhibit its expression. Following miR-140-5p directly targets PAK1 to inhibit its expression	80
HLX	Tumor promoter	Activation	HLX promote the transcription of PAK1	13
ELP3	Tumor promoter	Activation	ELP3 acetylates PAK1 at K420 to inhibit the dimerization of PAK1 which further promote its activity	101
LCAT1	Tumor promoter	Activation	LCAT1 up-regulates the activity of Rac1 to activate PAK1	88
Rac1	Tumor promoter	Activation	Rac1 phosphorylates PAK1 to activate its activity	86
CDC42	Tumor promoter	Activation	CDC42 phosphorylates PAK1 to activate its activity	86
Her2	Tumor promoter	Activation	Her2 leads to PAK1 recruitment and phosphorylation on Ser-423	2
cytoplasmic p27	Tumor promoter	Activation	cytoplasmic p27 phosphorylates PAK1 to activate its activity	96
STIL	Tumor promoter	Activation	STIL forms a ternary complex with ARHGEF7 and PAK1 and promote the phosphorylation of PAK1	111
PKC iota	Tumor promoter	Activation	PKC iota activates Rac1-PAK1 signalling	102
MLK3	Tumor promoter	Activation	MLK3 directly activates PAK1 kinase activity via phosphorylating PAK1 on Ser133 and Ser204 sites	95
NCK1	Tumor promoter	Activation	NCK1 enhances Rac1/PAK1 activity	104
RIT1	Tumor promoter	Activation	RIT1 interacts with PAK1 and CDC42/Rac1 to format complex and activates PAK1 signalling	103
P-REX1	Tumor promoter	Activation	P-REX1 activates Rac1/PAK1 pathway	105
Estrogen	Tumor promoter	Activation	Estrogen activates PAK1 through ERα and GPER1.	112
bFGF	Tumor promoter	Activation	bFGF activates PAK1 kinase activity via phosphorylation	97
CK2	Tumor promoter	Activation	CK2 activates PAK1 kinase activity via phosphorylation	98
PI3K	Tumor promoter	Activation	PI3K regulates the interaction of PAK1 with CK2α and CKIP-1 thus to activate its	98
CKIP-1	Tumor promoter	Activation	CKIP-1 recruits CK2 to PAK1 to increase its phosphorylation	98
JAK2	Tumor promoter	Activation	JAK2 activates PAK1 kinase activity via phosphorylation	99
PDK1	Tumor promoter	Activation	PDK1 phosphorylates PAK1 at Thr423 and activates its activity	100
BRAF	Tumor promoter	Activation	BRAF increased PAK1 expression and activity	113
Etk/Bmx	Tumor promoter	Activation	Etk directly associates with Pak1 via its N-terminal pleckstrin homology domain and also phosphorylates Pak1 on tyrosine residues.	114
BCAR3	Tumor promoter	Activation	BCAR3 augmentes the autophosphorylation and kinase activity of PAK1	115
JMJD6	Tumor promoter	Activation	JMJD6 increases the transcription of PAK1	116
Net1	Tumor promoter	Activation	Net1 dissociates and activates PAK1 dimers	117

**Table 2:** PAK1 inhibitors

No.	Comd.	Structure	Classification	PAK1 inhibition activity	References
1	K-252a	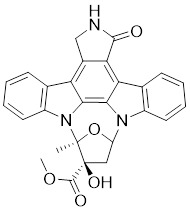	ATP-competitive inhibitors	2.4 nM	172
2	KTD606	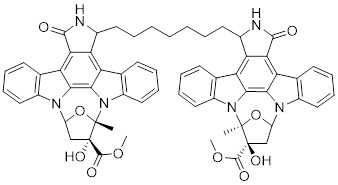	ATP-competitive inhibitors	4.0 nM	174
3	CEP1347	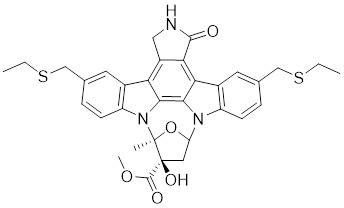	ATP-competitive inhibitors	2.5 nM	174
4	Staurosporine	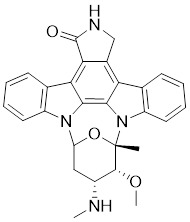	ATP-competitive inhibitors	0.75 nM	175
5	Λ-FL172	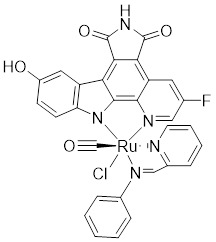	ATP-competitive inhibitors	130 nM	176
6	(*R*-1)	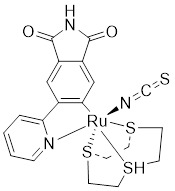	ATP-competitive inhibitors	83 nM	177
7	II-11	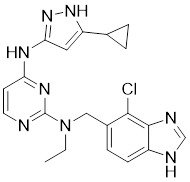	ATP-competitive inhibitors	1.6 nM	179
8	PF-3758309	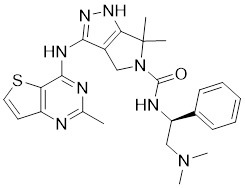	ATP-competitive inhibitors	14 nM	180
9	FRAX597	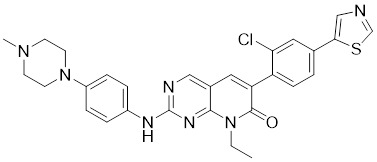	ATP-competitive inhibitors	7.7 nM	186
10	FRAX486	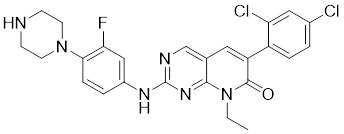	ATP-competitive inhibitors	8.3 nM	187
11	G‑5555	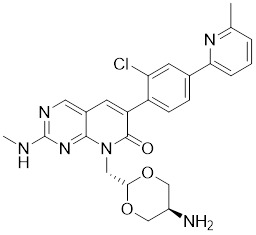	ATP-competitive inhibitors	3.7 nM	188
12	12	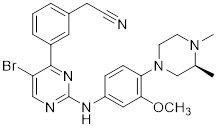	ATP-competitive inhibitors	65 nM	189
13	13	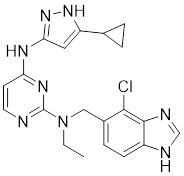	ATP-competitive inhibitors	5.0 nM	190
14	14	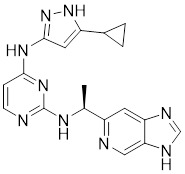	ATP-competitive inhibitors	5 nM	191
15	15	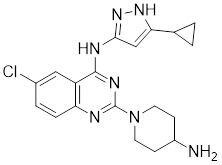	ATP-competitive inhibitors	288 nM	192
16	G-9791	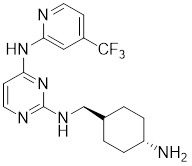	ATP-competitive inhibitors	Ki=26 nM	193
17	17	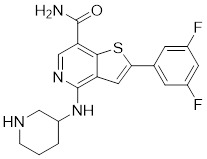	ATP-competitive inhibitors	0.73 μM	194
18	OSU-03012	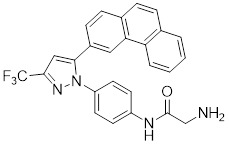	ATP-competitive inhibitors	1.03 μM	195
19	AK963	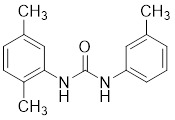	ATP-competitive inhibitors	-	196
20	ZMF-10	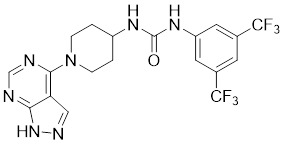	ATP-competitive inhibitors	194 nM	197
21	IPA-3	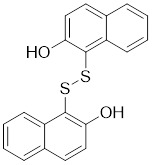	Allosteric inhibitors	2.5 μM	198
22	22	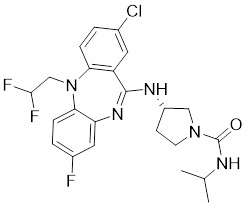	Allosteric inhibitors	5 nM	199
23	23	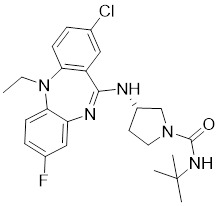	Allosteric inhibitors	18 nM	199
24	2-Mc-1,4-NHQ	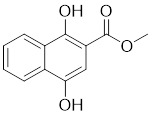	Allosteric inhibitors	-	200

## Abbreviations

PAK1: p21-Activated kinase 1
RAC1: Ras-related C3 botulinum toxin substrate 1
CDC42: Cell division control protein 42 homolog
MORC2: microrchidia CW-type zinc finger 2
PBD: p21-binding domain
AID: autoinhibitory domain
PIK3CA: Phosphatidylinositol 4,5-bisphosphate 3-kinase catalytic subunit alpha isoform
IL-1α: Interleukin-1 alpha
IL-6: Interleukin-6
IL-18: Interleukin-18
HIFs: hypoxia-inducible factors
HER2: Receptor tyrosine-protein kinase erbB-2
EGFR: Epidermal growth factor receptor
MAPK: Mitogen-activated protein kinase
EMT: epithelial-mesenchymal transformation
LIMK: LIM-kinase
TCoB: tubulin cofactor B
mTOR: Serine/threonine-protein kinase mTOR
miRNAs: microRNAs
LncRNA: long none-coding RNA
JAK2: Tyrosine-protein kinase JAK2
PPI: protein-protein interaction
GSK-3β: Glycogen synthase kinase-3 beta
MYC: Myc proto-oncogene protein
MMP: Matrix metalloproteinase
PPARγ: Peroxisome proliferator-activated receptor gamma
PIP2: phoshatidylinoside 4,5 bisphosphate
PIP3: phoshatidylinoside 3,4,5-triphosphate
MPNSTs: Malignant peripheral nerve sheath tumors
GHRH-R: hormone-releasing hormone receptor
DLC1: Dynein light chain 1
NIK: NF-κB interacting kinase
CDKs: cyclin-dependent kinases
PLKs: Polo-like kinases
Arpc1b: actin-related protein 2/3 complex subunit 1B
CHK2: checkpoint kinase 2
HTS: high-throughput screening
VS: virtual screening
FBDD: fragment-based drug design
SBDD: structure-based drug design
PROTAC: proteolysis targeting chimera
bFGF: basic fibroblast growth factor
VEGF: vascular endothelial growth factor
RUFY3: FYVE domain containing 3
ILK: Integrin-linked kinase
PDA: pancreatic ductal adenocarcinoma
PSCs: pancreatic stellate cells
PGM: Phosphoglucomutase 1
CML: chronic myeloid leukemia
HLX: H2.0-like homeobox
ELP3: elongator acetyltransferase complex subunit 3
MCAK: mitotic centromere-associated kinesin
GBM: glioblastoma
TGFβ: transforming growth factor beta
NSCLC: non-small-cell lung cancer
TGFBR1: transforming growth factor-β receptor type 1
BCR-ABL: BCR-ABL fusion gene
